# Oral metastasis of primary testicular choriocarcinoma: A case report and brief review of the literature

**DOI:** 10.4317/jced.63514

**Published:** 2025-11-30

**Authors:** Alexia Mariana Figueroa-Ramos, Raúl Hernández-Romero, Claudia H.S Caro-Sánchez, Guadalupe Teresa Hernández-Arana, Verónica Villatoro-Ugalde, Adalberto Mosqueda-Taylor

**Affiliations:** 1 Oral Medicine Clinic, Dermatology Division, General Hospital “Dr. Manuel Gea González”, Mexico City, Mexico; 2 Oral Pathology and Medicine Master’s Course, Health Care Department, Metropolitan Autonomous University, Xochimilco Unit, Mexico City, Mexico; 3 Head and Neck Tumors Department, National Cancer Institute, Mexico City, Mexico; 4 Pathology Department, National Cancer Institute, Mexico City, Mexico

## Abstract

A wide variety of neoplasms have the potential to metastasize to the oral region. Reported cases of testicular carcinoma are very rare, most of which have poor prognosis. When diagnosing tumors in patients with a previous history of carcinoma, it is important to consider the possibility of metastasis to the oral and maxillofacial region. However, in patients without a history of cancer, establishing the diagnosis represents a clinical challenge and requires multidisciplinary work. In this article, a case of testicular choriocarcinoma in the oral soft tissues is described, and a brief review of the literature is presented.

## Introduction

Approximately 1% of malignant neoplasms occurring in the oral cavity are metastatic lesions, which are generally indicative of advanced disease with a poor prognosis. In a significant proportion of cases, the primary carcinoma has already been diagnosed at the time of identifying the oral metastasis. However, it is estimated that in up to 25-30% of cases, oral lesions represent the first sign of the disease (1). Literature differs in terms of age and gender distribution (2), but some studies show a higher proportion of males among affected individuals in between the ages of 40 and 70 (3). In men the most common neoplasms that metastasize to the oral cavity are pulmonary, renal, hepatic and prostatic carcinoma, while in women breast, pulmonary, renal, colorectal and genital tumors are the most frequently identified (1 - 5). Any site in the oral cavity can be affected, but bone is more likely to be affected than oral mucosa (2:1 ratio). The mandible has been reported as the most common location (posterior area), while the gingiva represents the most frequently affected soft tissue site (2 , 3). In addition, there is some predilection for metastases to develop in certain oral sites, depending on the primary tumor, which seems to be related to the biological behavior of each pathological entity (2); as an example, carcinomas of prostate, mammary, adrenal, thyroid, and ocular origin have a predilection for bone tissue, while those of skin or stomach origin occur more commonly in the oral mucosa (4). The clinical appearance and symptoms of the lesions vary depending on the affected site. In bone tissue, for example, they may present as expansive and progressive growths, accompanied by symptoms such as paresthesia, pain and tooth loosening. While imaging characteristics are not specific, metastatic lesions mainly appear as radiolucent areas with ill-defined margins; however, they can also appear as radiopaque or mixed, and these characteristics may be related to the primary tumor and its osteoblastic or osteoclastic activity. In soft tissues metastases usually present as nodules that resemble benign neoplasms or reactive lesions (e.g. pyogenic granuloma or fibrous hyperplasia); these lesions are often asymptomatic and may or may not have an ulcerated surface (3 , 6). Treatment is complex and therapeutic options depend on factors such as tumor biology, site of origin and presence of other metastatic sites (3 , 4).

## Case Report

**Figure 1:**
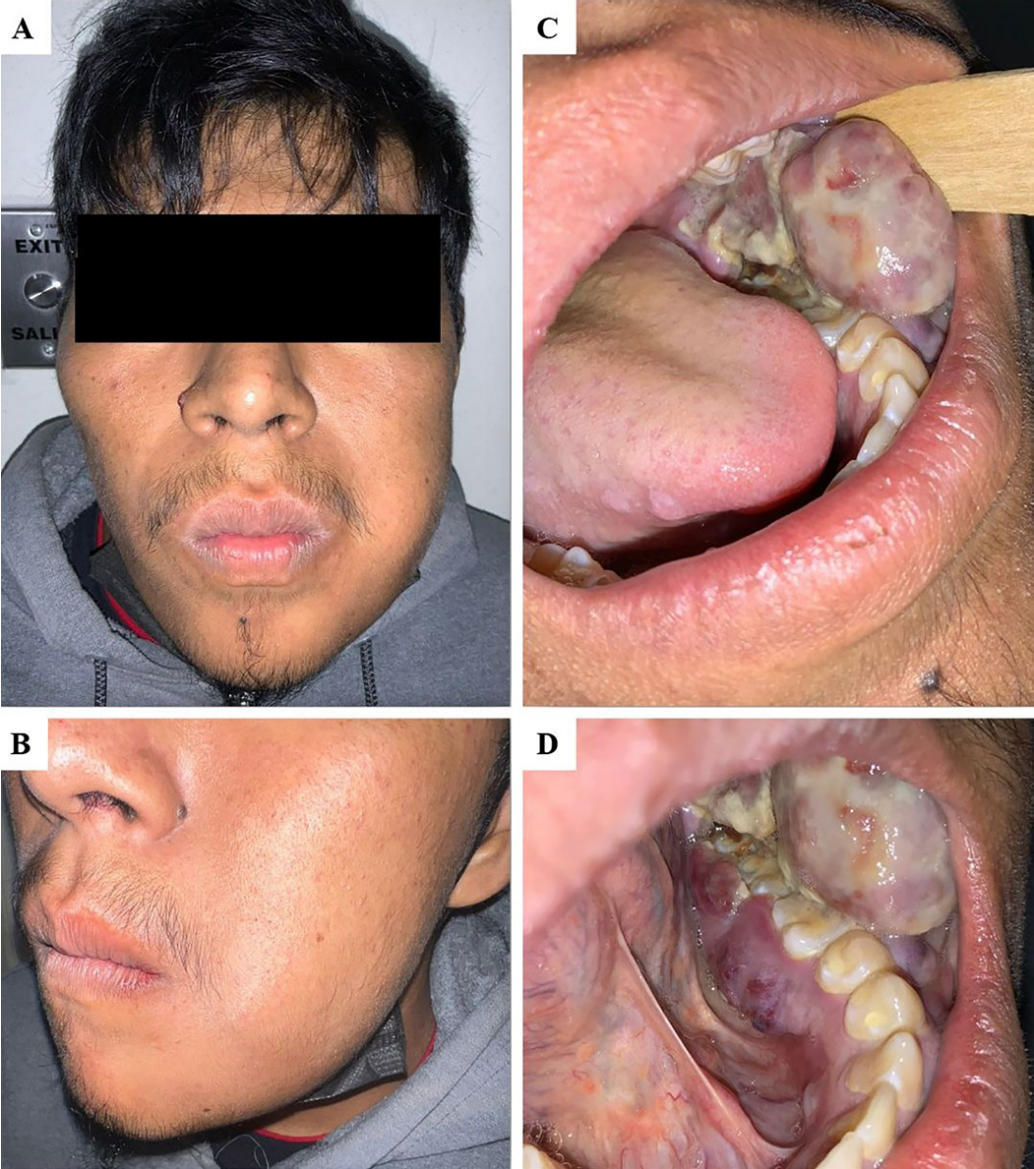
Extraoral and intraoral clinical findings. A and B) A tumor in the left buccal facial area is observed, altering the definition of the nasolabial sulcus. C) Intraorally examinations showed a purple-red multilobulated tumor located in left mandibular gingiva. D) The lesion extended from the premolar area to the retromolar trigone, along both the vestibular and lingual surfaces.

A 20-year-old male patient was referred by a dentist to the Oral Medicine Department of the General Hospital "Dr. Manuel Gea González" in Mexico City, for a painless intraoral tumor that had been present for 20 days. On clinical examination, the patient denied any significant medical history or drug use. He was conscious, well oriented, but with difficulty in speaking. Extraoral examination revealed a palpable tumor in the left buccal cheek area. The tumor was noted to be firm on palpation and slightly mobile, resulting in a disruption of the definition of the nasolabial sulcus (Fig. 1a,b).

1

A multilobulated, purple-red pedunculated tumor measuring 4 x 3 x 2 cm was identified intraorally on the left mandibular gingiva. The surface exhibited ulceration covered with fibrinoid pseudomembrane. It was also hemorrhagic, asymptomatic and did not cause paresthesia (Fig. 1c). The lesion partially covered the occlusal surfaces of the adjacent molars and extended from the second premolar to the retromolar triangle, along the lingual and vestibular surfaces, where it also showed erythematous areas (Fig. 1d).

**Figure 2:**
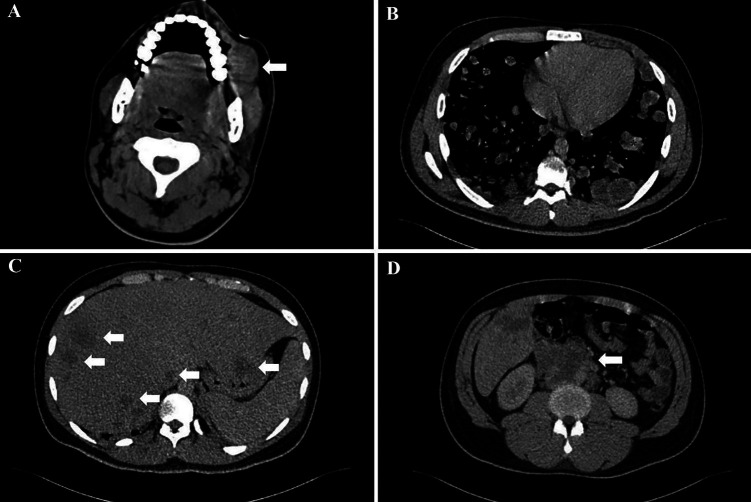
Imaging findings. A) Tumor located in the oral cavity involving soft tissues. It is in contact with the lower left molars (white arrow). B) Lung parenchyma with multiple bilateral metastatic lesions (red asterisks). C) Liver with multiple metastatic sites (white arrows). D) Retroperitoneal level with interaortocaval and lateral aortic adenopathies and involvement above the renal vessels (white arrow).

Orthopantomography revealed no osseous or dental alterations adjacent to the lesion. The differential diagnoses included a lymphoproliferative process, sarcoma and oral squamous cell carcinoma, without ruling out the possibility of metastasis of an unknown primary tumor. Due to the possibility of malignancy, the patient was referred to the National Cancer Institute, where computed axial tomography revealed an ovoid-shaped lesion in the oral cavity soft tissues, in close proximity to the lower left molars (Fig. 2a).

2

Aditionally, the patient exhibited metastatic activity in the lung parenchyma (Fig. 2b), liver (Fig. 2c), and retroperitoneal level (Fig. 2d). The presence of these patterns suggested the possibility of distant malignant neoplasm metastasis.

**Figure 3:**
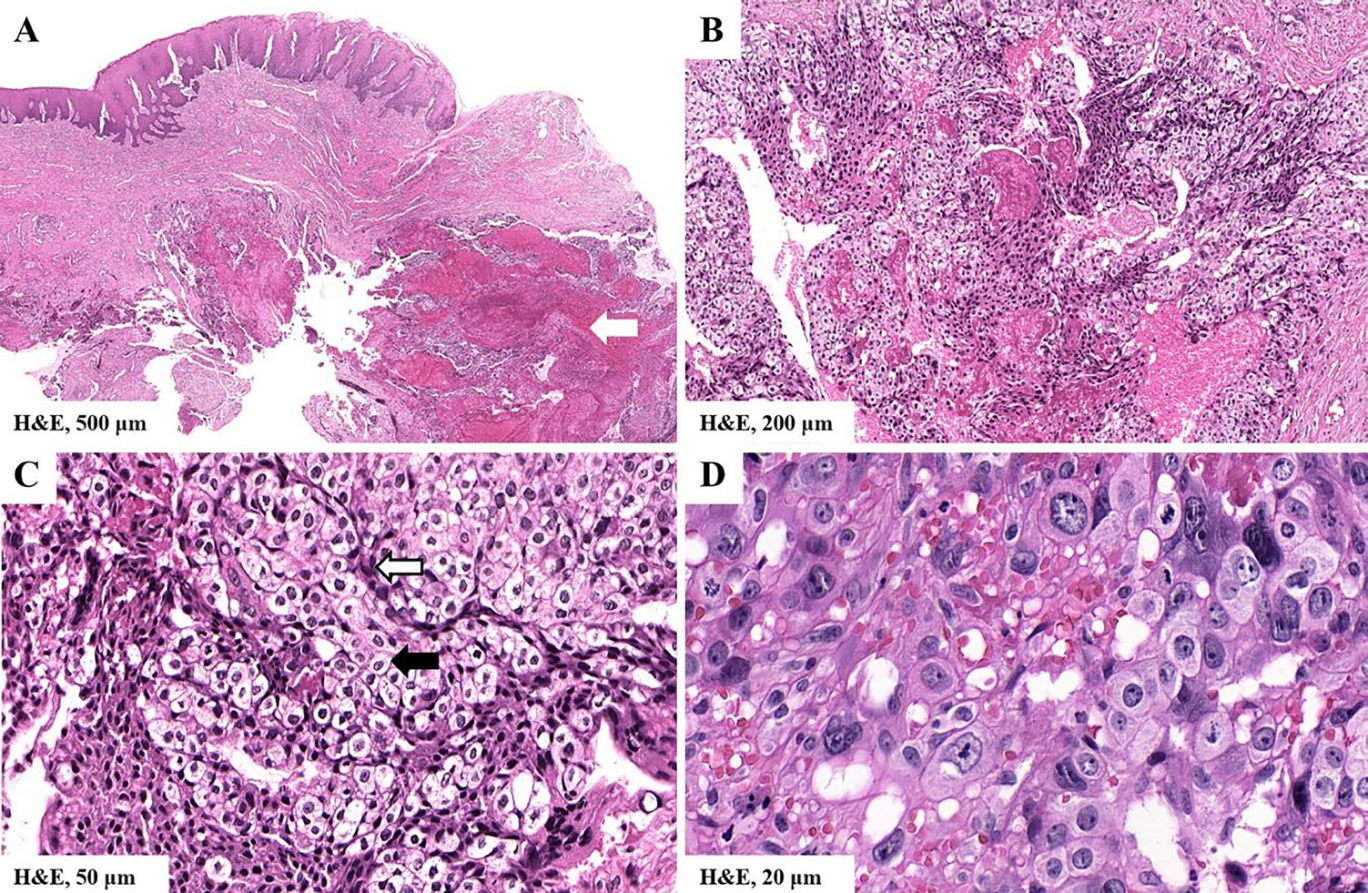
Histopathological findings. The slides were scanned and photomicrographs were obtained with the 3DHISTECH CaseViewer program. A) Mucosa fragment with extensive hemorrhagic and necrotic areas. B) Presence of neoplasia characterized by abundant cells arranged diffusely and in clusters. C) Biphasic cellular pattern consisting of conglomerates of polygonal mononuclear cells, with clear to eosinophilic granular cytoplasm, small to medium size (trophoblasts) (black arrow), surrounded by other spindle cells, with eosinophilic cytoplasm and hyperchromatic nuclei (syncytiotrophoblasts) (white arrow). D) Syncytiotrophoblasts showed severe cellular and nuclear pleomorphism, loss of cytoplasmic nuclear ratio, hyperchromatism, multinucleated cells and atypical mitoses.

A physical examination revealed the presence of a neoplasm in the right testicle, characterized by a firm consistency. The patient was not aware of this neoplasm, and therefore its evolution time remains unknown. A biopsy of the oral lesion was performed, and the patient was admitted for urgent oncological care. Tumoral markers were performed, yielding the following results: 181 ng/mL for alpha-fetoprotein (AFP) (normal: &lt;20 ng/mL/), 647,517 IU/mL for beta-subunit of human chorionic gonadotropin (HCG) (normal: &lt;20 IU/mL) and 2,322 IU/L for lactate dehydrogenase (LDH) (normal: &lt;150 IU/L). The incisional biopsy revealed a fragment of mucosa with extensive areas of necrosis, hemorrhage and abundant cells arranged diffusely and in small conglomerates adjacent to vascular spaces (Fig. 3a,b).

3

**Figure 4:**
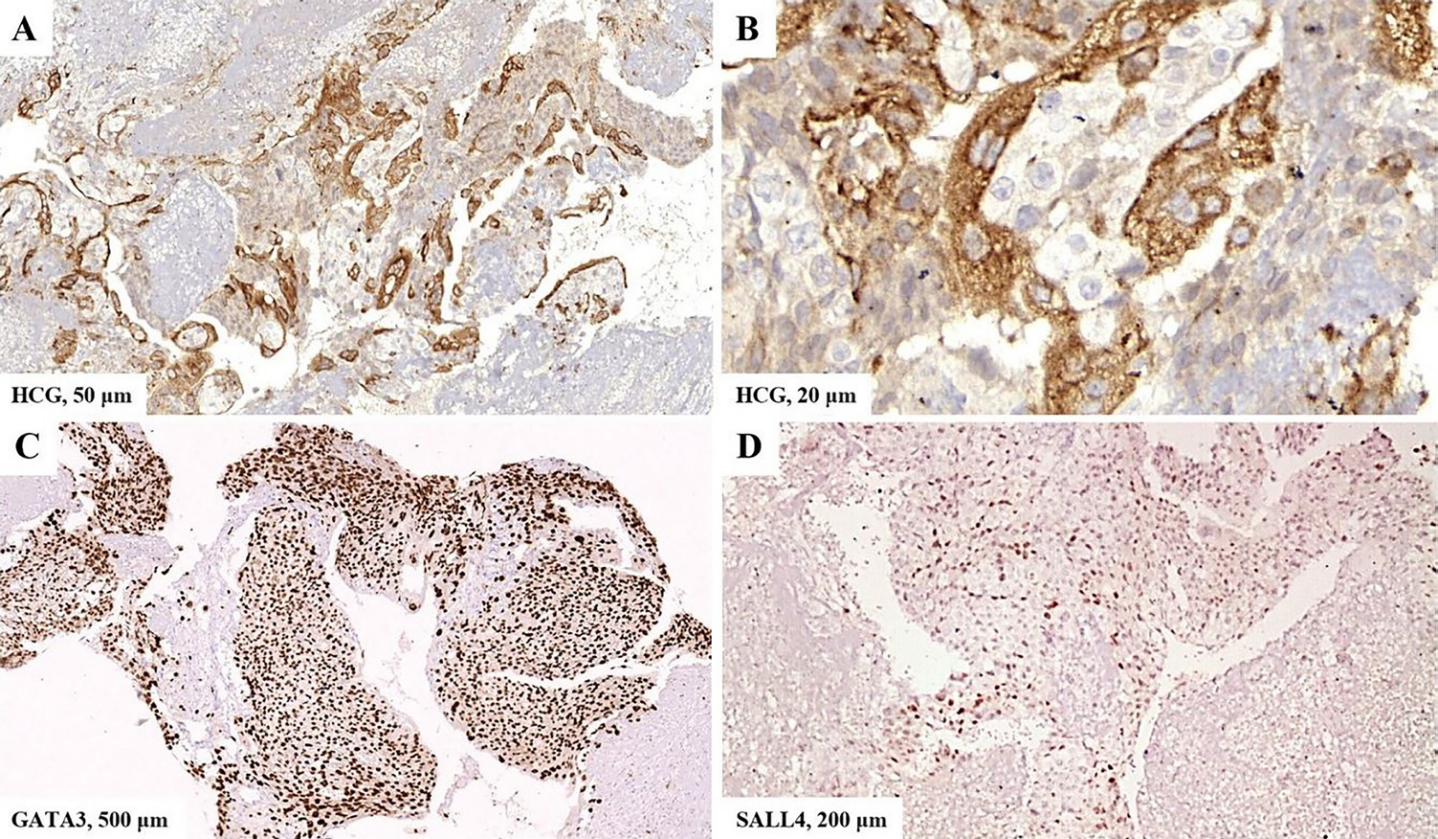
Immunohistochemistry reactions.The slides were scanned and photomicrographs were obtained with the 3DHISTECH CaseViewer program. A and B) Positive cytoplasmic labeling of HCG in syncytiotrophoblasts. C) Positive nuclear labeling of GATA3 in both cell types. D) Positive nuclear labeling of SALL4 in trophoblasts.

Upon higher magnification, a biphasic cellular pattern consisting of trophoblasts and syncytiotrophoblasts became apparent (Fig. 3c,d). Immunohistochemistry reactions showed positivity for HCG, GATA3 and SALL4 (Fig. 4) and negativity for OCT 3/4, CD30 and CD117.

4

The findings were consistent with the diagnosis of oral metastasis from germ cell carcinoma of the testis, pure choriocarcinoma subtype. Four days after hospital admission, systemic chemotherapy was started; unfortunately, the following day the patient developed alveolar hemorrhage, acute respiratory distress syndrome, multiorgan failure, and passed away.

## Discussion

The metastatic process involves several stages, including epithelial-mesenchymal transition, angiogenesis and intravasation, survival, proliferation and immune evasion; chronic inflammation has also been linked in the metastatic cascade due to its participation in multiple signaling pathways. The occurrence of metastatic deposits in the head and neck can be explained by the fact that malignant tumor cells evade pulmonary filtration through the valveless vertebral venous plexus, and the increased intrathoracic pressure directs blood flow to this region from the vena cava system (2 , 4). In relation to this type of neoplasm, it has been suggested that tumor emboli from the urogenital tract spread through the same plexus (7). Testicular cancer represents the most frequent malignant tumor in young men in more than sixty countries worldwide (8). The main risk factor is cryptorchidism (9), in addition to other causes such as pregnancy and birth complications, while genetic factors have been associated in less than 5% (10). Germ cell tumors constitute 95% of testicular tumors. According to their biological characteristics, these tumors are classified into two main categories: seminomas, which account for 50% of cases, and non-seminomas, which account the remaining 50%. Non-seminomas tumors are typically considered more aggressive (8 , 10), and can be classified as follows: mixed (33%), embryonal carcinomas (10%), teratomas (4%), yolk sac tumors (1%) and choriocarcinomas (0.3%) (9 , 11). Each of these subtypes exhibits distinct biological behavior and prognosis, hormone production, histopathology, and tumoral markers expression (12). Most patients present with a rapidly evolving and painless testicular tumor, with symptomatology related to metastatic disease (13). Testicular ultrasound is the initial investigative modality of choice in conjunction with a comprehensive medical history and physical examination. Tumor markers (HCG, AFP, LDH) should be requested, as well as a full body CT scan, while taking a transtesticular biopsy is not recommended, as it may guide drainage into the inguinal lymph nodes, and complicate treatment (10). Radical orchiectomy is usually the primary treatment in all cases, and depending on the stage of further disease, chemotherapy and/or radiotherapy is added (13). Nowadays, testicular carcinomas have been observed to exhibit one of the most favorable prognoses among malignant neoplasms in most developed countries (10). The metastatic spread of testicular germ cell neoplasms to the oral cavity is very infrequent, as there are only a few reported cases (2 , 14 , 15). Choriocarcinoma is the least common of all non-seminomatous germ cell tumors and has the worst prognosis due to its aggressive biological behavior (11 , 16). The disease manifests predominantly in males between the ages of 20 and 39, often accompanied by systemic symptoms associated with metastatic disease. It originates from trophoblastic tissue and consists of clusters of mononucleated trophoblasts (cytotrophoblasts and intermediate trophoblasts) and multinucleated syncytiotrophoblasts. It is generally observed to manifest in a mixed form, with a low percentage of cases presenting as a pure component. However, both presentations are unfavorable for the affected individuals. The diagnosis is usually made based on morphology, given the tissue's distinctive appearance, however, immunohistochemical reactions can be used to differentiate it from other germ cell tumors (17). The relevance of this case lies in the fact that very few cases of pure choriocarcinoma causing oral metastasis have been reported, and similar to our case, the prognosis has been quite unfavorable, with most cases culminating in death (16 , 18 - 20). Similar to the other reported cases, the tumor in our patient developed in the gingiva, which may be related to the microenvironment present in the chronically inflamed gingival tissue, acting as a favorable niche for colonization and proliferation of metastatic cells (4).

## Conclusions

In conclusion, the oral cavity is an important site for detecting both local and systemic diseases, including metastatic malignant neoplasms. Given its accessibility and the possibility of visual and tactile examination, it plays a crucial role in identifying manifestations that may otherwise go unnoticed in their initial stages. Therefore, the implementation of a thorough and systematic clinical oral examination, alongside a detailed and updated medical history, is essential for comprehensive patient care. Furthermore, the integration of multidisciplinary approaches - encompassing dental, medical, and oncological expertise - significantly enhances diagnostic accuracy and facilitates timely intervention, which in turn positively influences the prognosis and quality of life of affected individuals. Additionally, public health strategies should emphasize the promotion of self-examination and awareness campaigns for the early detection of neoplasms such as testicular carcinoma. These initiatives should receive similar attention to those targeting more prevalent carcinomas, as early recognition remains a cornerstone in improving survival rates and therapeutic outcomes across all types of malignancies.

## Data Availability

The datasets used and/or analyzed during the current study are available from the corresponding author.
